# Using Search Trends to Analyze Web-Based Interest in Lower Urinary Tract Symptoms-Related Inquiries, Diagnoses, and Treatments in Mainland China: Infodemiology Study of Baidu Index Data

**DOI:** 10.2196/27029

**Published:** 2021-07-06

**Authors:** Shanzun Wei, Ming Ma, Changjing Wu, Botao Yu, Lisha Jiang, Xi Wen, Fudong Fu, Ming Shi

**Affiliations:** 1 Department of Urology West China Hospital Sichuan University Chengdu China; 2 Andrology Laboratory West China Hospital Sichuan University Chengdu China; 3 Day Surgery Center West China Hospital Sichuan University China China

**Keywords:** lower urinary tract symptoms, patient education, Baidu Index, infodemiology, public interest, urinary tract disorders, infoveillance, web-based search, search engines, health care policy, digital health

## Abstract

**Background:**

Lower urinary tract symptoms (LUTS) are one of the most commonly described urination disorders worldwide. Previous investigations have focused predominantly on the prospective identification of cases that meet the researchers’ criteria; thus, the genuine demands regarding LUTS from patients and related issues may be neglected.

**Objective:**

We aimed to examine web-based search trends and behaviors related to LUTS on a national and regional scale by using the dominant, major search engine in mainland China.

**Methods:**

Baidu Index was queried by using LUTS-related terms for the period of January 2011 to September 2020. The search volume for each term was recorded to analyze search trends and demographic distributions. For user interest, user demand graph data and trend data were collected and analyzed.

**Results:**

Of the 13 LUTS domains, 11 domains are available in the Baidu Index database. The Baidu search index for each LUTS domain varied from 37.78% to 1.47%. The search trends for *urinary frequency* (2011-2018: annual percent change APC=7.82%; *P*<.001), *incomplete emptying* (2011-2014: APC=17.74%; *P*<.001), *nocturia* (2011-2018: APC=11.54%; *P*<.001), *dysuria* (2017-2020: APC=20.77%; *P*<.001), and *incontinence* (2011-2016: APC=13.39%; *P*<.001) exhibited fluctuations over time. The search index trends for *weak stream* (2011-2017: APC=−4.68%; *P*<.001; 2017-2020: APC=9.32%; *P*=.23), *split stream* (2011-2013: APC=9.50%; *P*=.44; 2013-2020: APC=2.05%; *P*=.71), *urgency* (2011-2018: APC=−2.63%; *P*=.03; 2018-2020: APC=8.58%; *P*=.19), and *nocturnal enuresis* (2011-2018: APC=−3.20%; *P*=.001; 2018-2020: APC=−4.21%; *P*=.04) remained relatively stable and consistent. The age distribution of the population for all LUTS-related inquiries showed that individuals aged 20 to 40 years made 73.86% (49,218,123/66,635,247) of the total search inquiries. Further, individuals aged 40 to 49 years made 12.29% (8,193,922/66,635,247) of the total search inquiries for all LUTS-related terms. People from the east part of China made 67.79% (45,172,031/66,635,247) of the total search queries. Additionally, most of the searches for LUTS-related terms were related to those for urinary diseases to varying degrees.

**Conclusions:**

Web-based interest in LUTS-related terms fluctuated wildly and was reflected timely by Baidu Index in mainland China. The web-based search popularity of each LUTS-related term varied significantly and differed based on personal interests, the population’s concerns, regional variations, and gender. These data can be used by care providers to track the prevalence of LUTS and the population’s interests, guide the establishment of disease-specific health care policies, and optimize physician-patient health care sessions.

## Introduction

Lower urinary tract symptoms (LUTS) are one of the most commonly described urination disorders worldwide [1]. There are a wide range of characteristic LUTS. These symptoms, such as urinary frequency, nocturia, urinary urgency, weak stream, hesitancy, terminal dribble, incomplete emptying, and urinary incontinence, can be categorized into three groups—storage, voiding, and postmicturition symptoms. These symptoms are generally manifestations of detrusor overactivity, sphincteric weakness, sensory bladder disorders, and prostate hyperplasia [1]. Due to the encompassing symptoms associated with sexual dysfunction and constant discomfort, LUTS significantly impair one's quality of life, social functioning, and workplace productivity and result in high health care costs [2-4]. The reported prevalence of LUTS is susceptible to numerous factors, such as diagnostic and assessment criteria (eg, International Continence Society [ICS] and The Expanded Prostate Cancer Index Composite study), evaluation tools (eg, International Prostate Symptom Score [IPSS] and Overactive Bladder Symptom Score), data collection, and various definitions, and therefore varies widely [5]. According to data from different multicenter surveys, the incidence of LUTS is between 26% and 86% [6-8]. In recent years, with the gradually heightened requirements for individuals’ quality of life and the incremental attention to the early symptoms of diseases, the public awareness of problems concerning LUTS has increased [9]. Similarly, the inconsistencies in previous LUTS prevalence investigations are noteworthy in China. Although these surveys were nationally conducted, the results can only provide the incidence of LUTS in specific populations—individuals aged above 18 years [10], males aged over 40 years [11], patients with benign prostatic hyperplasia (BPH) [12], and adult Chinese women [13]—for a sample size ranging from 3023 to 18,992. In some investigations, the annual LUTS incidents presented a growing trend, but patients' recognition and health care help-seeking rates were low [10-12]. It should be noted that previous investigations have focused predominantly on the prospective identification of cases that meet the researchers’ criteria. The data were collected with standardized questionnaires that were distributed via email, the agency's website, or on-site inquires [10,12]. Coupled with the lack of national surveillance and corresponding epidemiological reporting systems, the genuine demands regarding LUTS from patients and related issues may thus be neglected.

Nowadays, lives have been extensively changed by internet development. Internet searches have become peoples’ first-choice method for seeking information regarding health issues [14]. The advanced, mobile cyber technology used in emerging social media search engines have enabled the internet to become more accessible to people with all kinds of quests [15]. Additionally, artificial intelligence and big data technology enable search services to provide health-related and better suited information, especially to those who feel sick or suspect that they have early symptoms. It is believed that patients have been increasingly conducting web searches for health information prior to seeing a doctor. Hence, using data from Google has been successfully practiced when reporting the incidence of rhinitis [16], surveilling the prevalence of burn injuries [17], tracking e-cigarette–related lung injury cases [18], and forecasting and analyzing the public awareness of pandemic outbreaks [19].

In mainland China, Baidu has monopolized search services. After Google was forced to shut down its services in China, 92.1% of search volume data are now found in Baidu’s platform, and the usage of Baidu’s platform accounts for 93.1% of search service usage [20,21]. As the top-ranked search site [22], Baidu’s big data analyzing platform, Baidu Index, can reflect the genuine needs of the real world geospatially, temporally, and conclusively based on users’ specified terms [23-25]. It has been proven that Baidu Index is useful for surveilling and forecasting epidemic prevalence [23]. Additionally, the user behavior portrait may provide valuable insights into the health care concerns of populations and help with examining patients' experience sentiments [24]. These results will guide the tracking of common interests and the conduction of disease prevention and control education in a more focused manner [25].

In September 2020, the netizen population size reached 940 million in China [26]. For the first time, the China Internet Network Information Center has promoted the usage of internet health care services due to their better privacy and readiness. With 766 million users using the Baidu search service actively, its usage in relation to health inquiries and symptom confirmation have accounted for 63.16% of search service use [26,27]. Therefore, a pragmatic approach to monitoring LUTS incidents is analyzing data from the Baidu search index (BSI). The primary objective of this study was to assess the prevalence of LUTS by analyzing internet search activity and examining the validity of related topics. We also aimed to investigate the national demographics of people with LUTS and mass health-seeking concerns.

## Methods

### Keyword Selection and Data Retrieval

This study mainly analyzed the temporal search trends of LUTS-related terms in China. Symptoms domains were identified by referring to the ICS’s reports [1,5,28-30]. The Chinese terms were selected by referring to the ICS's LUTS translation recommendations to reduce results bias resulting from different language habits. All possible synonymous and derivative keywords for each term were screened [24,31] (Multimedia Appendix 1). Additionally, the availability of each keyword was examined on the Baidu Index platform. All available LUTS-related terms are listed in Multimedia Appendix 2.

In the trend analysis module, the search index value for each keyword is an absolute numerical value that is converted from normalized daily search counts. Hence, we obtained the search index values of each keyword from January 1, 2011, to September 12, 2020. All trend data were collected at the provincial and national levels [24,31]. For terms with multiple available keywords, we summarized the daily index value of all related keywords [25,31]. In addition to the trend data module, the keyword search-demand module shows the most noted correlating issues and sorts these issues by search frequency. The demographic portrait module records users’ ages and the regional distributions of inquired keywords. Therefore, we also collected user demand graph data and geodemographic data from the Baidu database to analyze netizens' demands and the public awareness of LUTS.

### Data Analysis

To analyze the search trend for each term, the corresponding BSI data were plotted sequentially to describe public attention. We calculated the medians and IQRs of daily search index values to describe annual and seasonal changes according to the normality and homogeneity of variance of the data. The definition of seasonal changes was taken from the standard definition provided by the National Meteorological Administration of China [32]. Statistical differences in index values from different periods were determined via the Mann-Whitney test. A Spearman correlation analysis was used to analyze the correlations among the search indices of common diseases with LUTS and LUTS-related terms. A *P* value of <.05 was considered statistically significant. The changes in trends over time for each domain was identified with the Joinpoint Regression model (Program Version 4.7.0.0; Statistical Research and Applications Branch, National Cancer Institute). This model is an ideal tool that is suited for examining big data over time and testing whether an apparent change in a trend is statistically significant. Annual percent change (APC) is the summary measure of a trend over a prespecified fixed interval [33].

### Statistical Analysis

All databases were constructed with Excel 2019 (Microsoft Corporation). The Mann-Whitney test and Spearman correlation analysis were conducted using IBM SPSS, version 22.0 (IBM Corporation). We used Prism 8 for macOS, version 8.4.0 (455) (GraphPad Software Incorporated) to conduct statistical analysis and create figures.

## Results

### Web-Based Data Trends of LUTS-Related Terms

**Figure 1:**
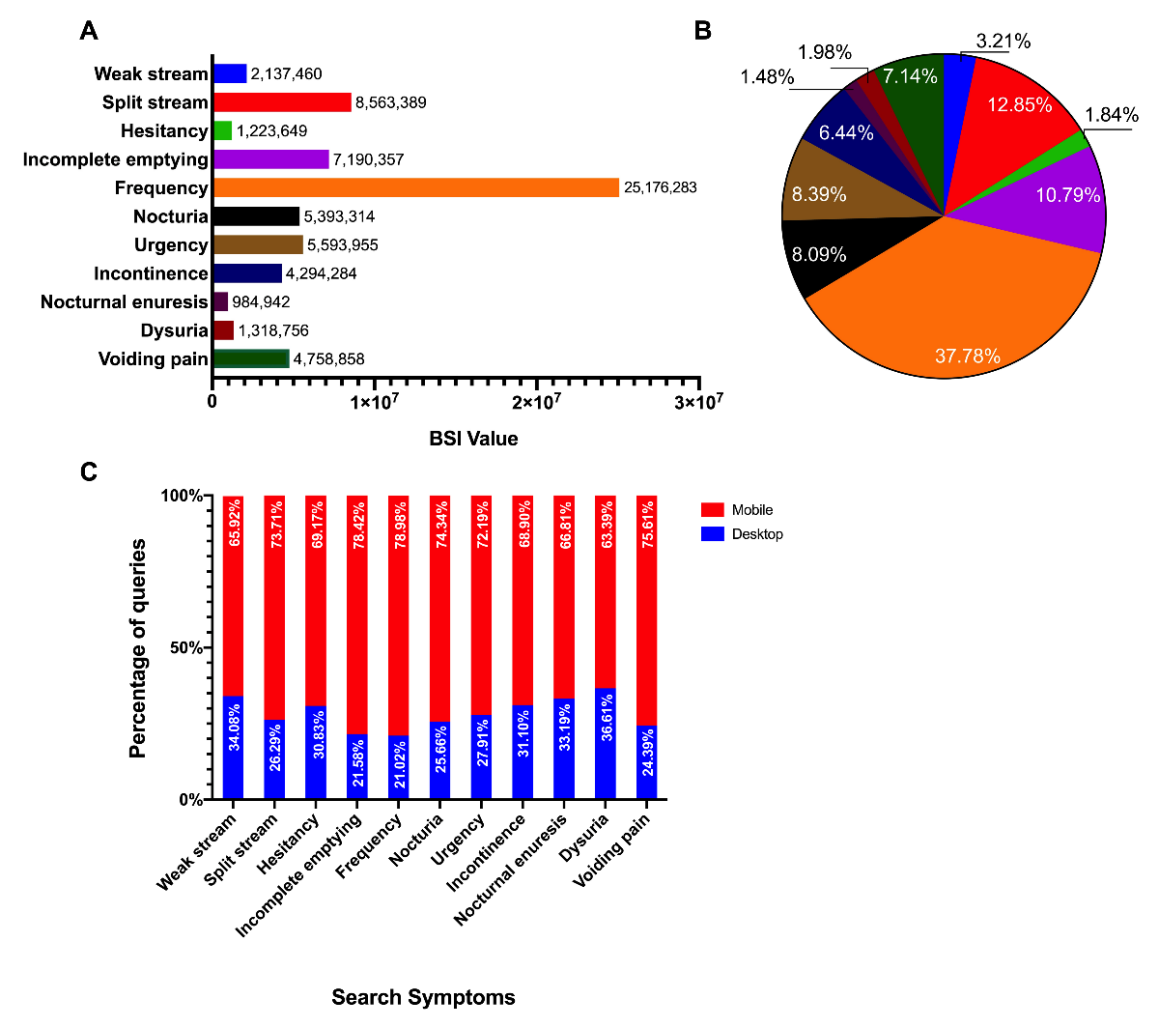
Web-based interest in LUTS domains. A: Total BSI value of each LUTS domain. B: BSI proportion of each LUTS domain. C: Ratio between mobile internet access and desktop access. BSI: Baidu search index; LUTS: lower urinary tract symptoms.

We summarized the total BSI values of LUTS-related terms from the past 10 years. Of the 13 LUTS domains, 11 domains were available in the Baidu Index database. The BSI for each LUTS term varied greatly. The term *urinary frequency* had the highest total BSI value (37.78%), whereas the term *nocturnal enuresis* only had a total BSI value of 1.47%. Additionally, we found that 75.31% (50,183,464/66,635,247) requests were from nondesktop computers, revealing that users were more likely to conduct searches with their mobile devices (Figure 1).

**Figure 2:**
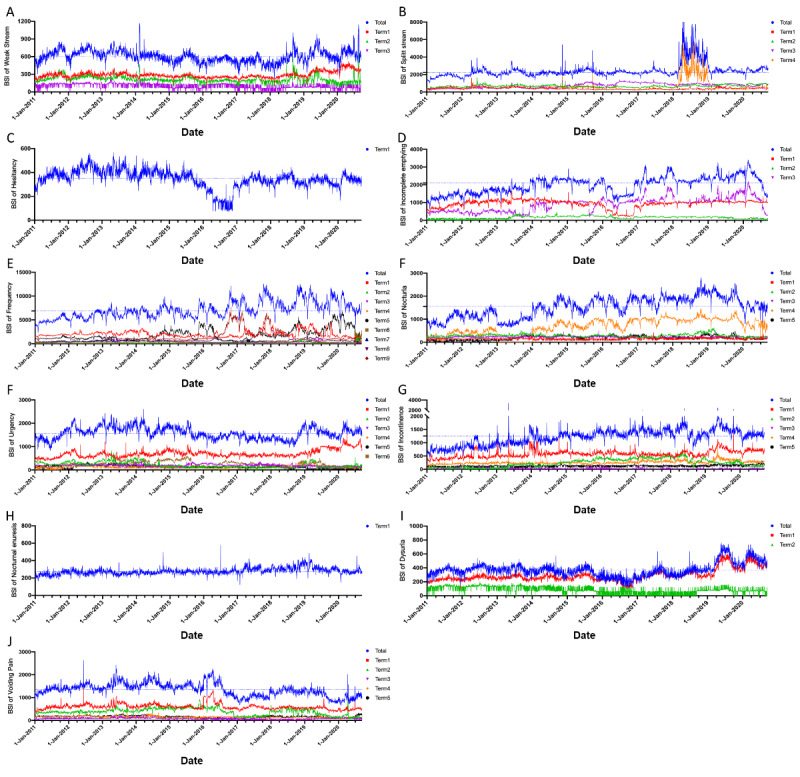
Real-time daily trends of web-based interest in each lower urinary tract symptoms–term over the last 10 years. BSI: Baidu search index.

We created a daily time series curve for the BSIs of each LUTS-related keyword for mainland China (Figure 2) and sorted the medians of each BSI value by year and season (Multimedia Appendix 3). Based on the median annual BSI for each LUTS-related term, the search trends for *urinary frequency* (2011-2018: APC=7.82%; *P*<.001), *incomplete emptying* (2011-2014: APC=17.74%; *P*<.001), *nocturia* (2011-2018: APC=11.54%; *P*<.001), *dysuria* (2017-2020: APC=20.77%; *P*<.001), and *incontinence* (2011-2016: APC=13.39%; *P*<.001) relatively grew for a period of time. The search index trends for *weak stream* (2011-2017: APC=−4.68%; *P*<.001; 2017-2020: APC=9.32%; *P*=.23), *split stream* (2011-2013: APC=9.50%; *P*=.44; 2013-2020: APC=2.05%; *P*=.71), *urgency* (2011-2018: APC=−2.63%; *P*=.03; 2018-2020: APC=8.58%; *P*=.19), and *nocturnal enuresis* (2011-2018: APC=3.20%; *P*<.001.; 2019-2020: APC=−4.21%, *P*=.44) remained relatively stable and consistent (Multimedia Appendix 4). A notable spike in search volume was found for the term *split stream* in the year of 2018, and this mainly resulted from a surge in searches for term 4 (*尿尿分叉*; ie, *Wee Wee split*). With regard to the terms *hesitancy* and *voiding pain*, an annual downward trend was found for each keyword. With regard to seasonal differences, though the trends for *frequency* (*P=*.057), *incontinence* (*P=*.36), and *nocturia* (*P=*.27) seemed to fluctuate in a pattern, the seasonal BSI gap for each term was not significant.

### Geographic Differences

**Figure 3:**
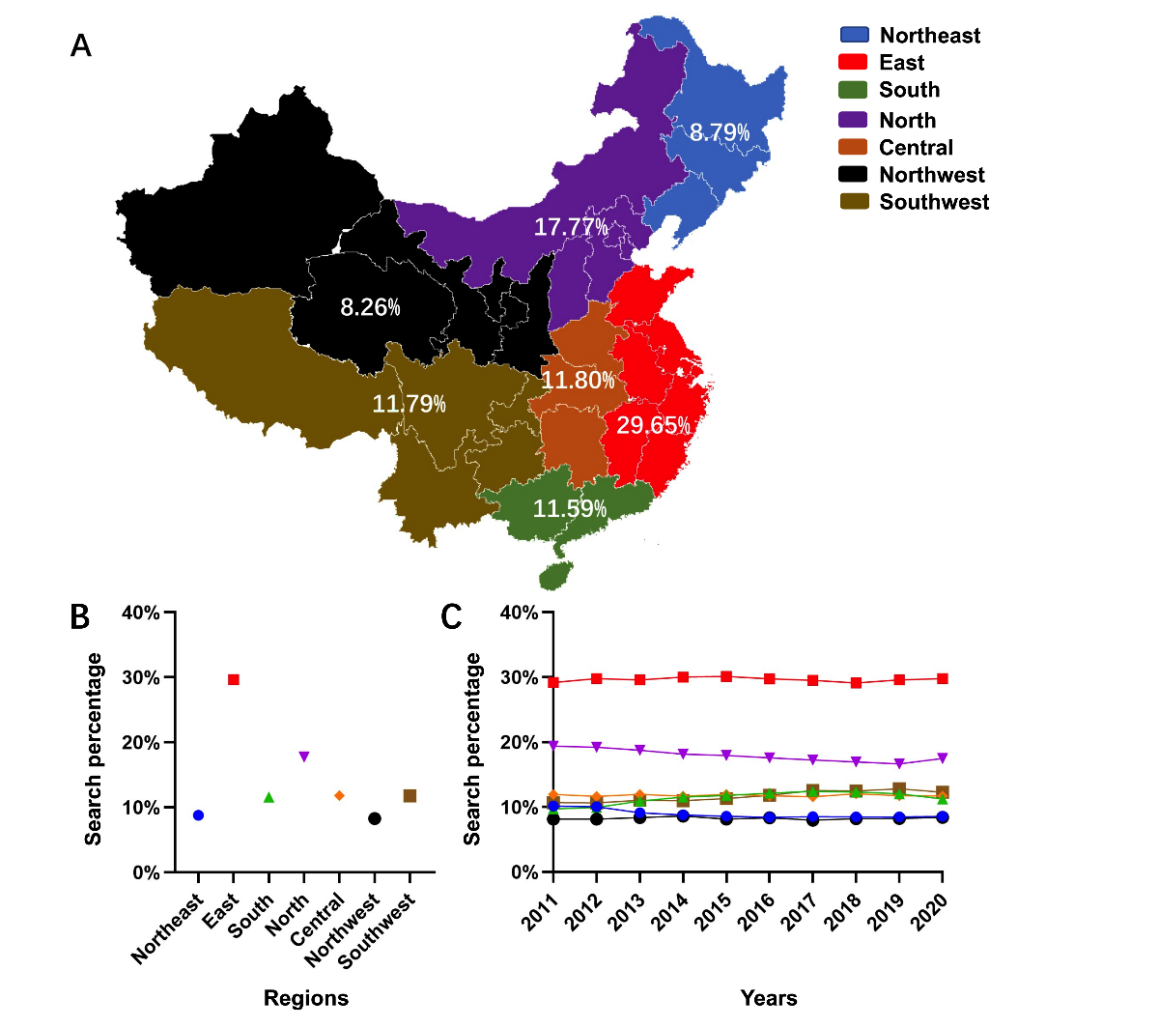
Regional distribution of web-based interest based on lower urinary tract symptoms–related searches over the last 10 years (with available data). A: Regional rates for each area. B: 10-year total search rates for each area. C: Annual trend of the Baidu search indices for each region.

Geographic differences in LUTS-related terms’ BSI data were calculated based on provincial data and sorted according to Chinese administrative divisions. These regions were Northeast China, East China, South China, North China, Central China, and Northwest and Southwest China. In Figure 3, the 10-year regional BSI proportions for all LUTS-related terms are presented in a map of mainland China. Additionally, the regional BSI proportions for each year are presented. It was notable that people from the east part of China (East, North, Northeast, and South China) made 67.79% (45,172,031/66,635,247) of the total search queries. However, the queries from West China (northwest and southwest) only accounted for 20.05% (13,360,352/66,635,247) of the search queries. The regional BSI proportion of each LUTS-related term and the annual trends are presented in Multimedia Appendix 5.

### Demographic Differences

**Figure 4:**
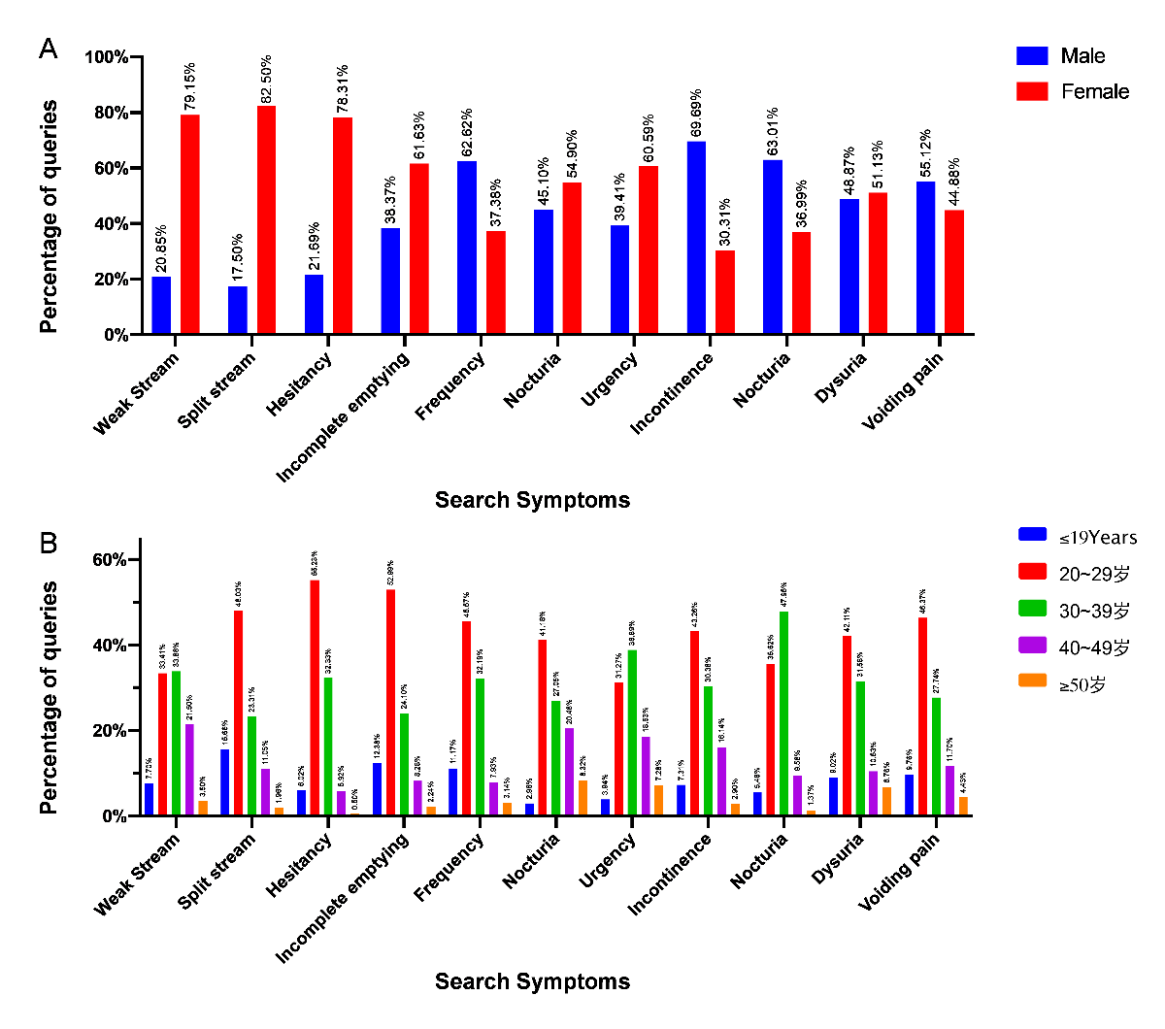
Demographic distributions of lower urinary tract symptoms–related searches. A: Gender distribution. B: Age distribution.

The age distribution of the population for all LUTS-related inquiries showed that individuals aged 20 to 40 years made 73.86% (49,218,123/66,635,247) of the total search inquiries. Further, individuals aged 40 to 49 years made 12.29% (8,193,922/66,635,247) of the total search inquiries for all LUTS-related terms. People aged over 50 years accounted for the least amount of general search inquiries. With regard to gender differences, searches for the term *urinary frequency*, *incontinence*, *nocturia*, and *voiding pain* were more prevalent in male populations. Other terms, such as *weak stream*, *split stream*, and *hesitancy*, were mainly inquired by female populations (Figure 4).

### Search Incentives

LUTS are chronic conditions that are common in adult men and are frequently associated with BPH, chronic prostatitis, cystitis, urethritis, and urinary tract infection. Hence, we examined the correlation among the daily BSIs of these terms in order to explore people’s motivation for conducting LUTS-related searches. It was revealed that most of the searches for LUTS-related terms were related to those for urinary diseases to varying degrees (Table 1). There was no significant correlation between BPH and *weak stream* (*P*<.11) and between chronic prostatitis and *dysuria* (*P*=.053).

**Table 1:** Correlations between lower urinary tract symptoms–related search terms and related diagnoses (N=3543).

Search terms	Benign prostatic hyperplasia		Chronic prostatitis			Cystitis			Urethritis			Urinary tract infection	
	*r*	*P* value	*r*	*P* value	*r*		*P* value	*r*		*P* value	*r*		*P* value
Weak stream	0.011	.11	−0.240	<.001	0.344		<.001	0.098		<.001	−0.106		<.001
Split stream	0.527	<.001	0.230	<.001	0.313		<.001	0.490		<.001	0.664		<.001
Hesitancy	−0.218	<.001	−0.219	<.001	0.324		<.001	−0.145		<.001	−0.287		<.001
Incomplete emptying	0.636	<.001	0.378	<.001	0.064		<.001	0.443		<.001	0.608		<.001
Nocturia	0.657	<.001	0.407	<.001	0.139		<.001	0.325		<.001	0.659		<.001
Dysuria	0.222	<.01	−0.033	.053	0.197		<.001	0.255		<.001	0.159		<.001
Incontinence	0.707	<.001	0.053	<.001	0.061		<.001	0.531		<.001	0.677		<.001
Urinary frequency	0.624	<.001	0.492	<.001	0.086		<.001	0.386		<.001	0.598		<.001
Nocturnal enuresis	0.498	<.001	0.305	<.001	0.330		<.001	0.196		<.001	0.519		<.001
Urgency	0.071	<.001	0.083	<.001	0.273		<.001	0.085		<.001	−0.188		<.001
Odynuria	−0.305	<.001	−0.291	<.001	−0.359		<.001	−0.166		<.001	−0.279		<.001

### Relative Terms of Keywords and Search Frequency

**Figure 5:**
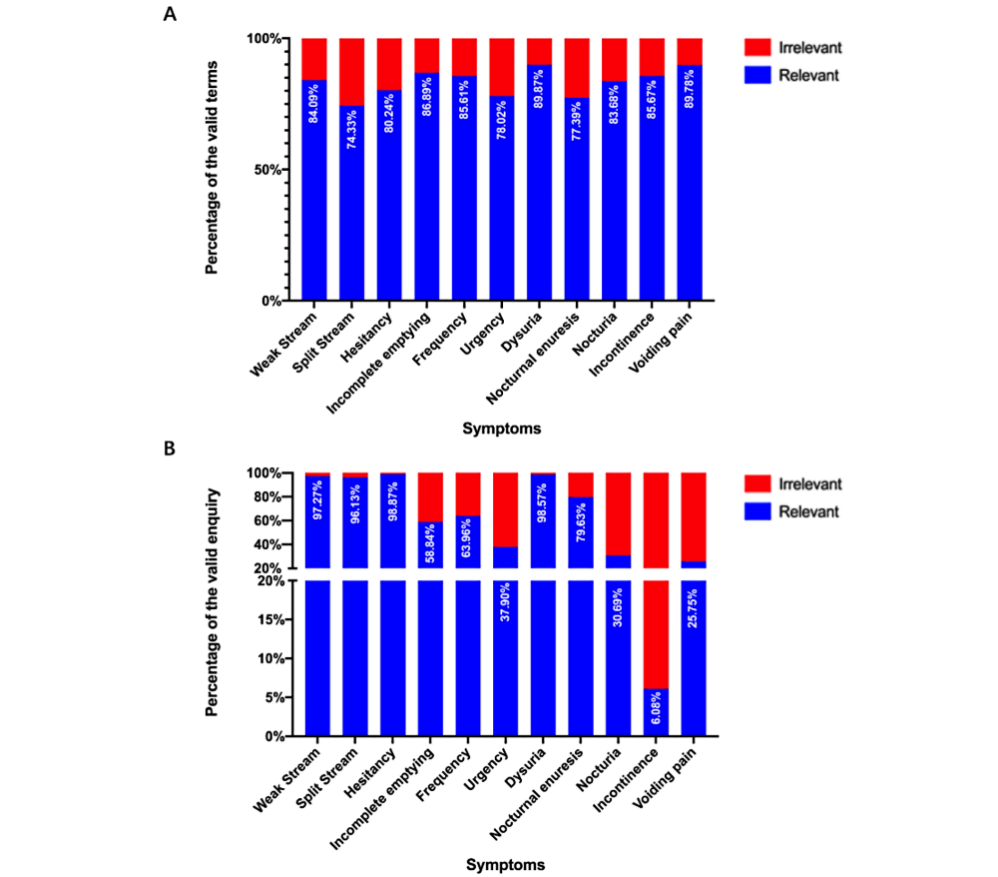
The percentage of valid searches for lower urinary tract symptoms–related terms. A: The percentage of valid term inquiries. B: The frequency of valid term inquiries.

**Figure 6:**
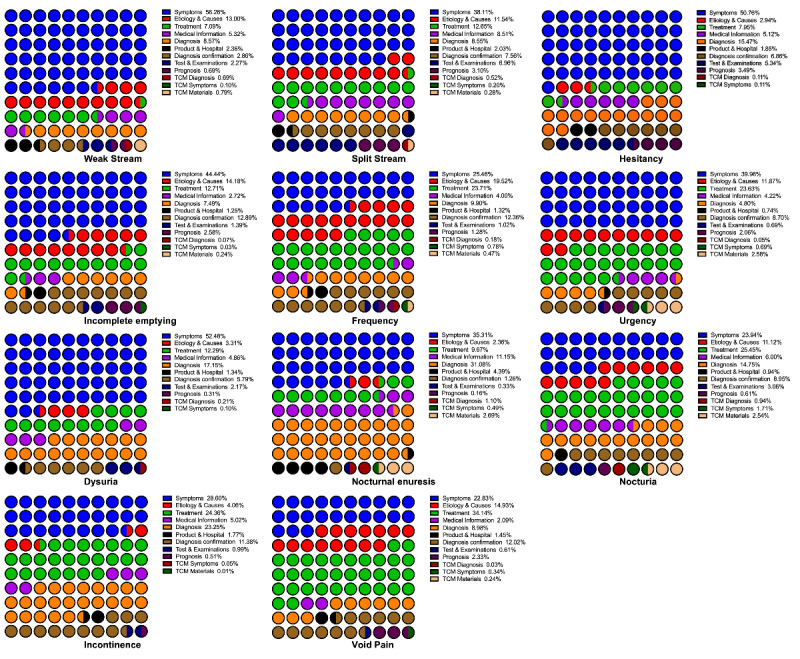
The percentage of term inquiry categories in Baidu Index related to lower urinary tract symptoms. TCM: traditional Chinese medicine.

**Figure 7:**
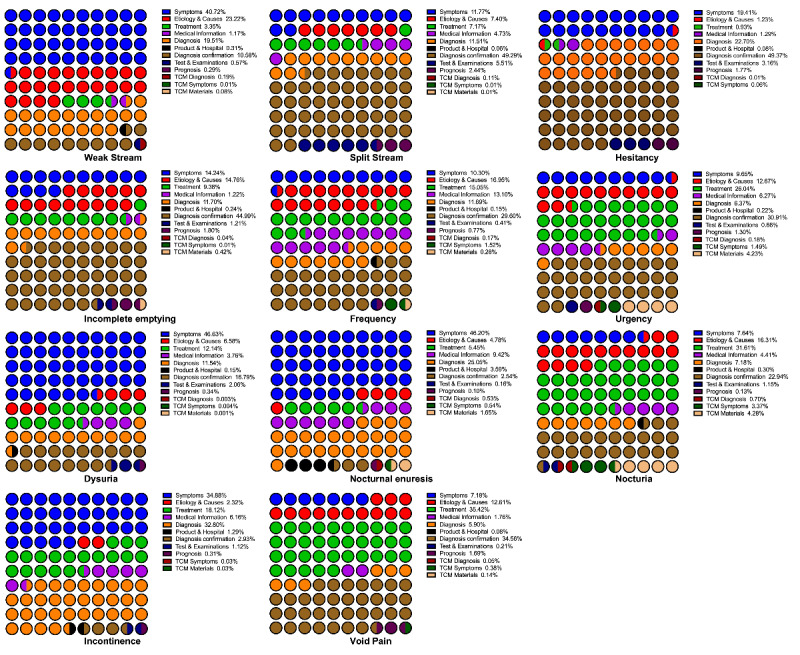
Percentage of enquiry frequency categories related to LUTS in Baidu Index. LUTS: Lower Urinary Tract Symptoms, TCM: Traditional Chinese Medicine.

We reviewed and sorted the top searched relative terms of keywords in the Baidu Index platform. In order to classify the relative terms based on the main concerns of the users, 13 hypothetical (used to describe users’ concerns more explicitly and comprehensively) categories of domains and their core meanings were defined. These categories were as follows: irrelevant, symptoms, ethology quests, treatment, medical information, diagnoses, products and hospitals, diagnosis confirmation, tests and examinations, prognosis, traditional Chinese medicine–conceptualized quests related to diagnoses, symptoms, and treatment materials. Detailed percentages of the relevant search terms and search frequencies are listed in Figure 5. In Figure 6 and Figure 7, the inquiry terms and frequencies related to LUTS in Baidu Index are categorized and presented with their percentages. Furthermore, the top 3 terms for each domain are listed in Multimedia Appendix 6, along with their BSIs.

## Discussion

### Principal Findings

Our findings revealed that internet search trends in Baidu Index could comprehensively reflect the actual concerns of Chinese populations regarding health issues. Previous studies have confirmed that users' information-seeking behavior profiles from big data platforms are adeptly proficient in forecasting disease outbreaks, identifying interest at the population level, monitoring health education campaigns, and tracking the trends of patients' preferences [14,15,24,25]. As a primary health care information source, patients are more inclined to initiate a preliminary consult on the internet before seeing a doctor [34]. Hence, investigations of the infodemiology and infoveillance features of LUTS-related terms are capable of revealing a massive amount of users’ behavioral information and reflecting actual prevalence trends to some extent [24,25]. This feature is particularly crucial when there are a lack of related epidemiology data and real-time information.

By using Baidu Index, we found that the popularity of each entry varied greatly. The most inquired term, *urinary frequency*, was searched 25 times more often than the least inquired term, *nocturnal enuresis*. Intuitively, this result shows different degrees of attention for LUTS and therefore reflects the relative prevalence of symptoms in the population. With regard to trends, we illustrated that search volumes for each LUTS-related term had maintained a certain order of magnitude. A sustained growing trend was observed for the terms *frequency*, *incomplete emptying*, *nocturia*, and *incontinence*. The trends for other phrases were relatively flat and steady. These trends indicate that while the prevalence of LUTS may remain stable, public awareness and internet penetration may be on the rise, and people’s habits of seeking medical advice are changing. Further, 75.31% (50,183,464/66,635,247) of the search requests were conducted on mobile devices. This fact may have resulted from the development of portable technology and mobile internet. Some clinics use smartphones to track older adults with frailty and their quality of life [35]. Additionally, 43.5% of individuals have acknowledged that they use cellphones while on the toilet; therefore, it is no trouble for these users to conduct searches while “on the go,” especially when they are experiencing a LUTS attack [36].

We noticed a spike in search volume for the term *incontinence*, which surged to over 3400 from 1100 on May 6, 2013. However, just 15 days prior, an earthquake (7.0 on the Richter scale) struck Ya'an, Sichuan Province, resulting in 196 deaths, 21 missing individuals, and 11,470 injured individuals. We believe that the spike in search volume for *incontinence* is related to this event, as most incontinence symptoms would have appeared after the injuries from the earthquake [37]. Additionally, we noticed an abnormal fluctuation that was above the average search volume for the term *split stream*. The total increase in this term’s search volume originated from term 4 (*尿尿分叉*; ie, *Wee Wee split*). We suspect that this may have been caused by the bidding manipulation of specific keywords by some individuals or institutions because the popularity of other terms has not increased significantly [25]. However, due to the openness of the Baidu Index platform, this abnormal fluctuation can only affect the search popularity of a specific term for a certain period of time [23]. Nevertheless, it will not affect the popularity of other synonyms. Additionally, despite the fact that the competitive ranking promotion mechanism is well known in Baidu's product line, this mechanism is limited to ranking search results instead of guiding or changing users' search preferences by shifting their needs. Hence, this abnormal search volume phenomenon only occurs occasionally. The relatively consistent search trend for other terms suggests that the BSI can still be a good indicator of public attention toward specific LUTS if keywords are sufficiently included in the search.

After analyzing the regional differences in search volumes, we found that the search trends of each LUTS-related term were in line with those of other infodemiology research [24,25]. The search volume for each LUTS-related term was highest in the East China region, followed by those in North, South, and Central China. In Northwest China, search volumes were lowest. However, the trends revealed by the BSI contradict previous investigations on the prevalence of LUTS and BPH. In earlier investigations, the prevalence of LUTS was highest in the northwest and northeast regions and lowest in the southwest areas [12,38]. However, at the provincial level, the prevalence of LUTS in Guangdong, Shanghai, and Beijing was ranked highest compared to those of other provinces [38]. These three provinces are representative of the South, East, and North China regions. Further, the data sets of previous investigations pooled individuals aged over 40 years. As a result of this, the rankings from these investigations were not much different from ours. Moreover, medical infrastructures, internet access, and public health awareness are considered better in these higher income regions. Coupled with the fact that Baidu's data sources involve people of all ages from across the whole country, the geographic patterns in BSIs may reflect the socioeconomic and population rankings of regions in mainland China.

In Groutz et al’s [39] and Heylen et al’s [40] investigations, the prevalence of voiding difficulty in the female population was vastly underestimated. The prevalence of voiding difficulty significantly increases with age and the degree of pelvic organ prolapse [39,40]. Additionally, voiding symptoms such as weak streams, hesitancy, and strained urination are especially prevalent in females when other comorbidities are present [41]. Specifically, the pooled incidence rate of voiding difficulty is 21.3% and 15.5% in males and females, respectively [42]. Thus symptom differences based on sex are not significant. Further, when comparing LUTS prevalence rates, Apostolidis et al [43] recognized that they failed to investigate incidents of incontinence in both sexes. This was because certain symptoms, such as incontinence, are not listed in the IPSS or National Institutes of Health Chronic Prostatitis Symptom Index assessments, which are the most commonly applied assessments and checklists for men with LUTS and chronic pelvic pain syndrome symptoms [42]. The IPSS is universally used, even when evaluating the efficacy of prostate surgery—a known leading cause of male urinary incontinence [44,45]. The evaluations for male incontinence is listed in the “expanded” index for “prostate cancer patients” [46]. This is probably because of the belief that male incontinence has almost always resulted from prolonged bladder outlet obstruction [47]. Nevertheless, it should be noted that other causes, such as sphincter injury following prostatic surgery, polypharmacy, detrusor degeneration, and sustained urinary tract infection, have also been identified as common factors of male incontinence onset [48]. Therefore, prostate problems may be an essential factor of the systematic neglect of primary incontinence symptoms in the male population. Similarly, the diagnostic criteria for female bladder obstruction have been misestimated, and it is recommended that female bladder obstruction should be confirmed with urodynamic examinations [39,43]. Despite these issues, there are differences in the distribution of LUTS between men and women. It is worth pointing out that the real factor that determines whether patients seek medical treatment is not the characteristics of symptoms but the severity of symptoms [49]. Additionally, as internet users with web-based health issue inquiries are 60% more likely to seek and use health care services, the ratio of BSIs for reported symptoms could be used to remind practitioners to avoid ignoring “uncommon” symptoms in each gender population [50].

Baidu Index lists the top 10 related theme words for each search term. In this platform, the trends for correlated search terms are calculated and updated weekly. This system enables practitioners to gain insight into the most concerning problems from patients, confirm individuals’ main intentions, and examine the most exposed information to users. In our study, we found that the related theme words were not limited to the field of disease diagnosis, cause, and treatment, as previously described [25]. Instead, users had an extensive range of questions and concerns about LUTS (eg, the best hospital, treatment efficacies, and disease prognosis), and Baidu Index includes a lot of content that has nothing to do with health counseling. The differences in the distribution of the theme words varied vastly for each LUTS-related term. In terms of the relevance ratio, the term *dysuria* had the highest number of relevant term entries and search frequencies. However, although the term *urinary incontinence* was included in 85.67% (8134/9495) of the number of valid search entries, this term had the lowest search volume (1,726,303/283,932,352, 6.08%). Further, we noticed that the terms related to symptoms, etiology, and treatment were the terms that were the most inquired by Baidu platform users. With regard to the term *incontinence*, a large number of inquiries related to diagnosis mainly included the term *incontinence*, which is both a symptom description and a diagnostic. Hence it is clear that Baidu platform users are more inclined to make inquiries by describing their symptoms and seek treatment. Although the diagnosis and prognosis of LUTS are also of concern, the rate is relatively low.

We also noticed that there were numerous inquiries that were formatted as “what are the symptoms of...?” This form of question implies that Baidu platform users may have a definite awareness of the diagnoses related to their symptoms and that they have needed to confirm or exclude a particular diagnosis. This is a typical internet self-assessment pattern. Further, for the treatment category, we found that most users focused on inquiries such as “the most effective medication” or “the fastest efficacy.” These facts reveal that Baidu platform users’ views of LUTS and related diagnoses are one-sided. Their intention is to control LUTS as soon as possible instead of consulting a doctor to obtain a definite diagnosis and treatment regimen. It is worth pointing out that despite the fact that diseases indicated by LUTS are often not life-threatening, completing routine urine tests or ultrasonic examinations as part of standardized diagnosis and treatment procedures can at least reduce the likelihood of misdiagnosing malignancies [51,52]. Furthermore, some patients believe that examinations with negative results are conducted with the purpose of making a profit and are therefore meaningless and a waste of money [53]. These beliefs may worsen when these patients come across information that claims people can cure LUTS with free, “do-it-yourself” methods. Such information will strengthen their prejudice against hospitals and increase their likeliness of using “do-it-yourself” methods to heal their LUTS [54]. Patients’ limited understanding of health issues predisposes them to misdiagnoses, results in illness delays, and poses considerable health risks.

### Limitations

Several limitations in this study should be addressed. One is that Baidu Index only analyzes search data from Baidu and does not analyze search data from social media platforms. Therefore, the data generated by these platforms could not be assessed. Further, the types of Baidu platform users could not be determined due to confidentiality (ie, users’ privacy protection). Consequently, the analysis of demographic data regarding information-seeking preferences and behaviors could only be based on age, gender, and regions. Information such as socioeconomic status, ethnicity, and educational background were not obtainable. Furthermore, LUTS and their associated diseases, such as prostatitis, BPH, and cystitis, are not covered by national surveillance systems and lack corresponding epidemiological data. Additionally, instead of real search frequencies, the BSI is just a weighted index derivative; we could only speculate the relative popularity of LUTS-related search terms and their development trends.

Notwithstanding these limitations, this is the first study that investigates public concerns about LUTS, to the best of our knowledge. We chose to examine the infodemiology characteristics of LUTS because the clinical manifestations of LUTS are obvious to patients. As such, LUTS are easy to compare when analyzing clinical diagnoses and diseases. Therefore, research on the search volumes of LUTS-related keywords can directly mirror existing problems from the patient perspective.

Finally, we believe that infodemiology research based on the BSI reveals people's behaviors when they search for related keywords and that such data can be used as an important reference for understanding the population’s needs. In terms of medical and health-related information, Baidu should seek government health management departments' help to revise and standardize medical-related keywords and consultations, so that their platform can better serve consumers, potential patients, health management departments, and medical practitioners. Since the real-time data on search volumes and information-seeking behaviors on the platform are renewed over time, these data can potentially be used to provide supplementary references and up-to-date information for improving care practice standards and making policies in a timelier manner.

### Conclusion

Web-based interest in LUTS-related terms fluctuated wildly and was reflected timely by Baidu Index in mainland China. Significant variability was observed in the web-based search popularity of each LUTS-related term, and popularity differed based on personal interests, the population’s concerns, regional variations, and gender differences. These data can be used by medical professionals to track the prevalence of LUTS and the population’s interests, guide the establishment of disease-specific health care policies, and optimize physician-patient health care sessions.

## Appendix

Multimedia Appendix 1 Flowchart of symptom domains and term confirmation. BSI: Baidu search index; ICS: International Continence Society; LUTS: lower urinary tract symptoms.

Multimedia Appendix 2 List of keywords used in the composite search index.

Multimedia Appendix 3 Web-based interest in lower urinary tract symptoms domains over the last 10 years.

Multimedia Appendix 4 Joinpoint graphic of Baidu search index trends for each each lower urinary tract symptoms domain.

Multimedia Appendix 5 Regional distribution of web-based interest for each lower urinary tract symptoms domain over the last 10 years. A: Regional rates for each area. B: Annual Baidu search index trends for each region.

Multimedia Appendix 6 List of the most inquired terms for each lower urinary tract symptoms domain.

## Abbreviations

APC: annual percent change
BPH: benign prostatic hyperplasia
BSI: Baidu search index
ICS: International Continence Society
IPSS: International Prostate Symptom Score
LUTS: lower urinary tract symptoms
