# Supplementary figures and images for: Using Search Trends to Analyze Web-Based Interest in Lower Urinary Tract Symptoms-Related Inquiries, Diagnoses, and Treatments in Mainland China: Infodemiology Study of Baidu Index Data
Source: J Med Internet Res. 2021 Jul 6;23(7):e27029. doi: 10.2196/27029 (PMC8292938; doi:10.2196/27029)

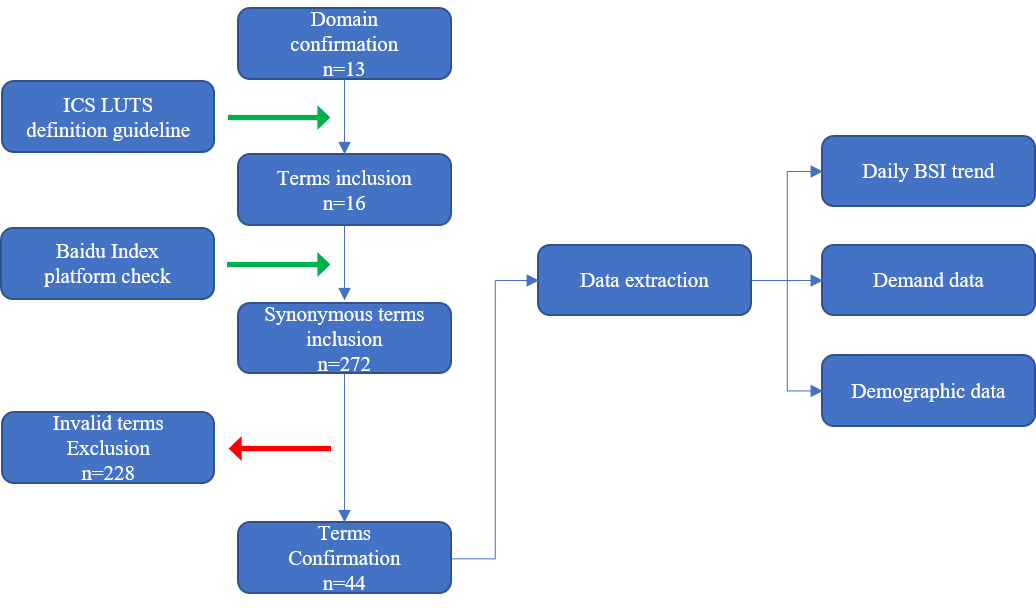

Supplement: Multimedia Appendix 1 [file jmir_v23i7e27029_app1.png]

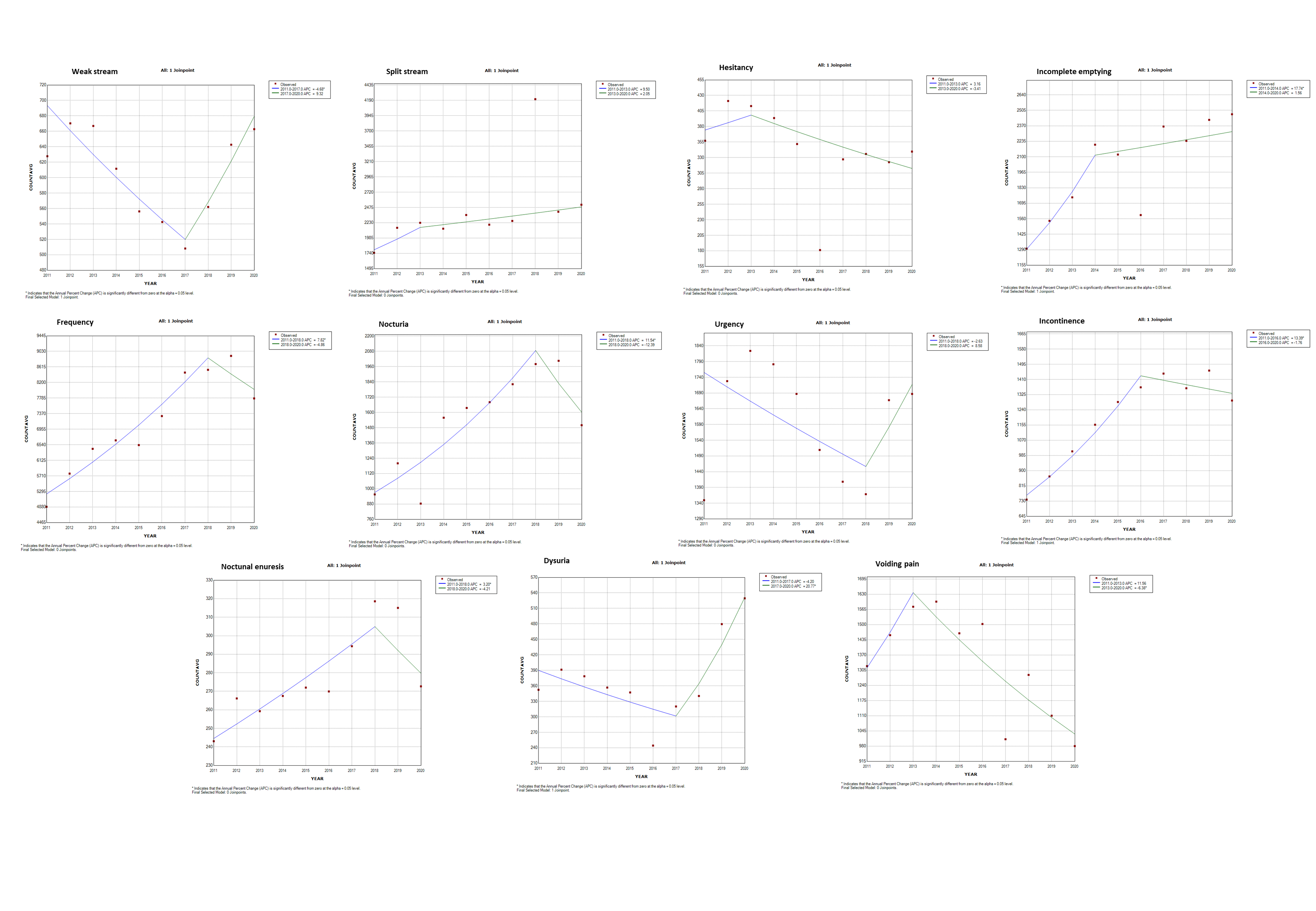

Supplement: Multimedia Appendix 4 [file jmir_v23i7e27029_app4.png]
