# Supplementary material for: Using Search Trends to Analyze Web-Based Interest in Lower Urinary Tract Symptoms-Related Inquiries, Diagnoses, and Treatments in Mainland China: Infodemiology Study of Baidu Index Data
Source: J Med Internet Res. 2021 Jul 6;23(7):e27029. doi: 10.2196/27029 (PMC8292938; doi:10.2196/27029)
Supplement: Multimedia Appendix 2 [file jmir_v23i7e27029_app2.pdf]

List of keywords used in composite search index

Term and recommend translation in Chinese	Available term in Search engine	English equivilent terms	Code
Weak stream<br>尿流缓慢	尿无力	Weak urination when peeing	Term1
	小便无力	Weak in urine voiding	Term2
	排尿无力	Weak in voiding urination	Term3
Split stream<br>尿流分叉或喷洒状尿流<br>（描述性症状）	小便分叉	Split stream in voiding	Term1
	尿分叉	Split stream when peeing	Term2
	尿分叉是怎么回事	What is with the split stream	Term3
	尿尿分叉	Wee Wee split	Term4
Intermittency<br>间歇性尿流（尿流间歇）	NA		
Hesitancy<br>排尿踌躇	尿等待	Waiting before peeing	Term1
Straining<br>用力排尿	NA		
Terminal dribble<br>终末滴沥	NA		
Perceived frequency<br>日间排尿次数增加	尿频	Urinary frequency	Term1
	尿频是什么原因	The cause for urinary frequency	Term2
	尿频的原因	Urinary frequency’s cause	Term3
	尿频是什么原因导致的	What causes urinary frequency	Term4
	尿频是怎么回事	What is with urinary frequency	Term5
	尿频吃什么药	What medicine should take to treat urinary frequently	Term6
	尿频怎么办	What to do with urinary frequency	Term7
	尿频尿急	Urinary frequency with urgency	Term8
	尿频尿急是怎么回事	What is urinary frequency with urgency	Term9
Nocturia<br>夜尿	夜尿增多	Night urinartion increasion	Term1
	夜尿多	Increased night urination	Term2
	夜尿	(Increased) night urination	Term3
	夜尿多是怎么回事	What is with increased night urination	Term4
	夜尿多吃什么药	What medicine should take to treat increased night urination	Term5
Urgency<br>尿急	尿急	Urinary urgency	Term1
	尿急是怎么回事	What is with urinary urgency	Term2
	尿急怎么办	What to do with urinary urgency	Term3
	尿急吃什么药	What medicine to take	Term4
	尿急尿频尿痛	Urinary urgency with frequency and voiding pain	Term5
	尿急尿频尿不尽	Urinary urgency with frequency and	Term6
Incontinence<br>尿失禁	尿失禁	Urinary incontinence	Term1
	尿失禁的治疗方法	Treatment for urinary incontinence	Term2
	尿失禁的护理	Nursery for urinary incontinence	Term3
Stress incont. (laughing, sneezing,coughing)<br>压力性尿失禁	压力性尿失禁	Stress incontinence	Term4
Urgency incont. (with fear of leaking)<br>急迫性尿失禁	急迫性尿失禁	Urgency incontinence	Term5
Stress incont. (physical activities)<br>混合性尿失禁	NA		
Leak for no reason<br>持续性尿失禁	NA		
Post-micturition incontinence	NA		

排尿后尿失禁			
Leak during sexual activity （Conditional） 其他类型的尿失禁	NA		
Nocturnal enuresis 遗尿	遗尿	Nocturnal enuresis	Term1
Incomplete emptying 未排空感	尿不尽	Incomplete urinary emptying	Term1
	尿不尽症状	Symptoms of incomplete urinary emptying	Term2
	尿不尽是怎么回事	What is with incomplete urinary emptying	Term3
Dysuria 排尿困难	排尿困难	Difficult urination	Term1
	女性排尿困难	Female difficult urination	Term2
Voidingpain 排尿疼痛	尿痛	Urinary pain	Term1
	尿痛是怎么回事	What is with urinary pain	Term2
	尿痛是什么原因	What is the cause for urinary pain	Term3
	尿痛怎么办	What to do with urinary pain	Term4
	尿痛吃什么药	What medicine to take with urinary pain	Term5
