# Supplementary material for: Using Search Trends to Analyze Web-Based Interest in Lower Urinary Tract Symptoms-Related Inquiries, Diagnoses, and Treatments in Mainland China: Infodemiology Study of Baidu Index Data
Source: J Med Internet Res. 2021 Jul 6;23(7):e27029. doi: 10.2196/27029 (PMC8292938; doi:10.2196/27029)
Supplement: Multimedia Appendix 3 [file jmir_v23i7e27029_app3.pdf]

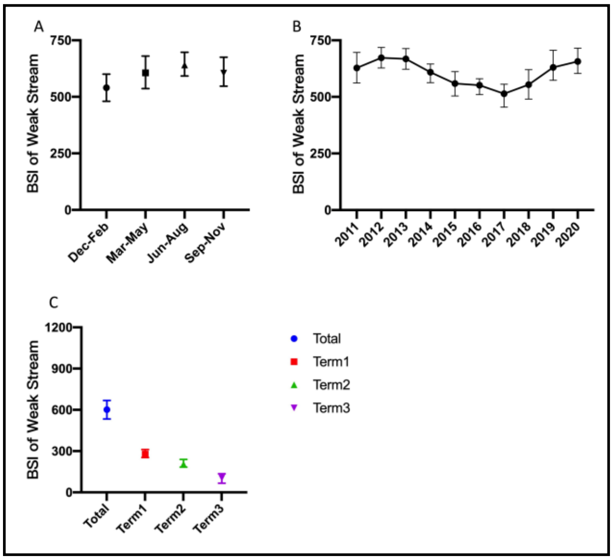

Weak stream

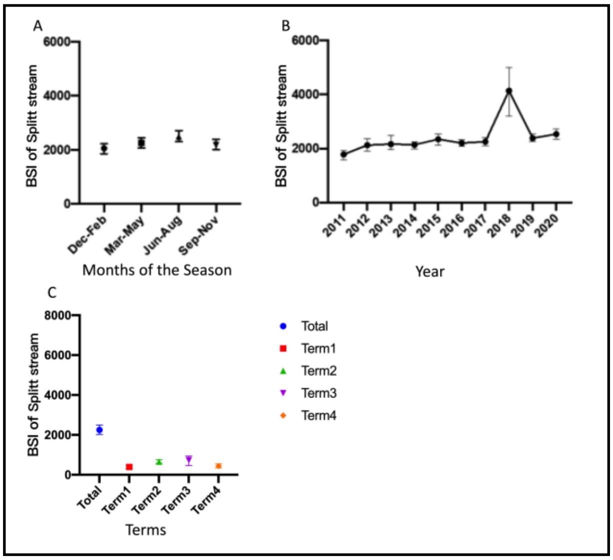

Split stream

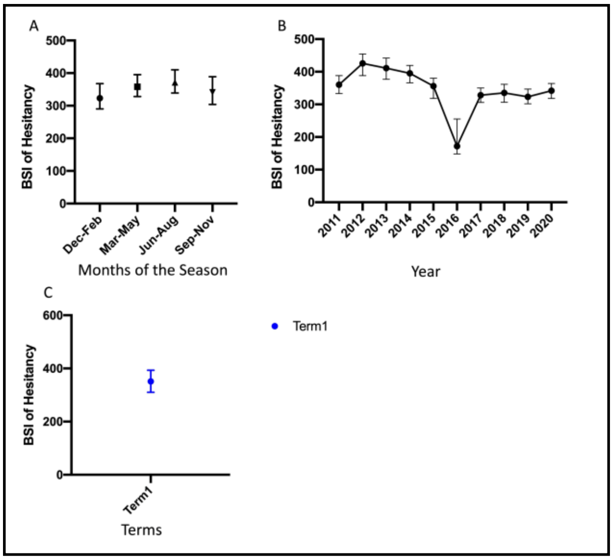

Hesitancy

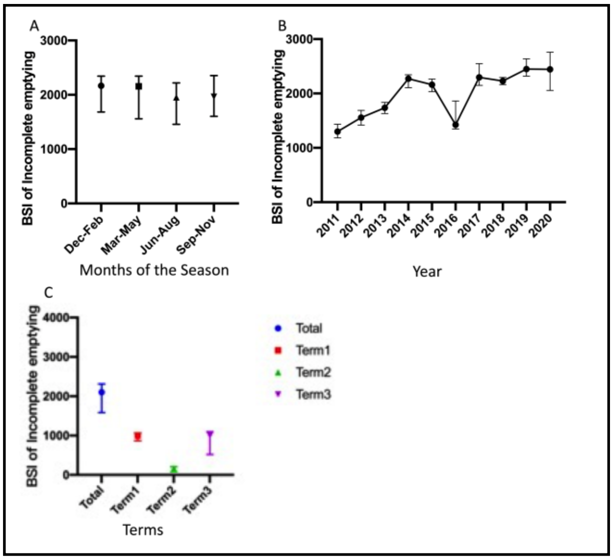

Incomplete emptying

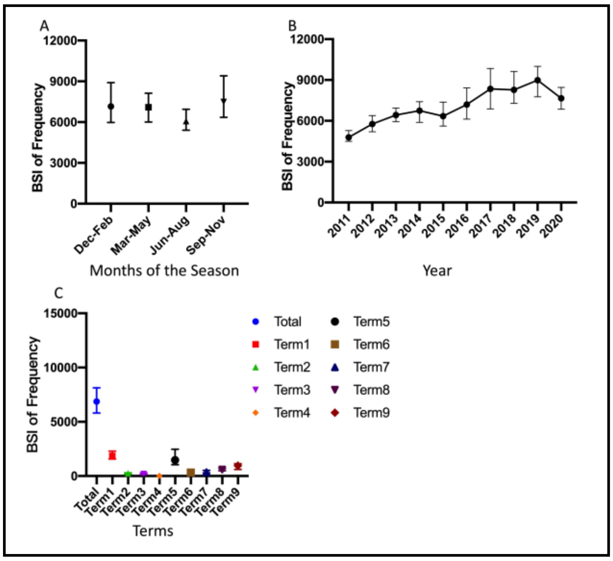

Frequency

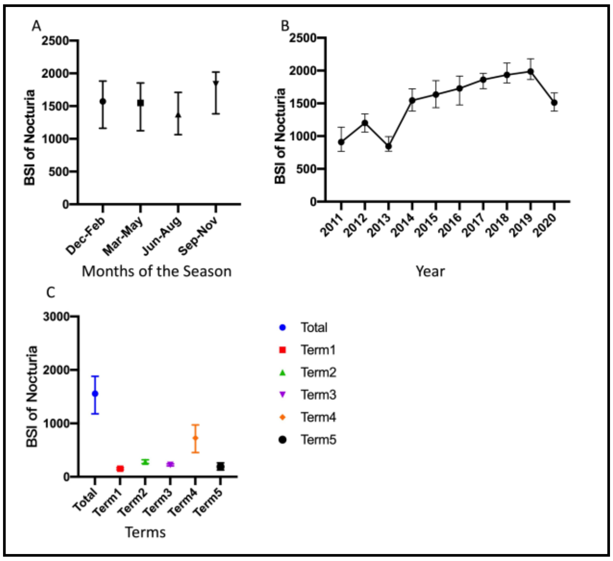

Nocturia

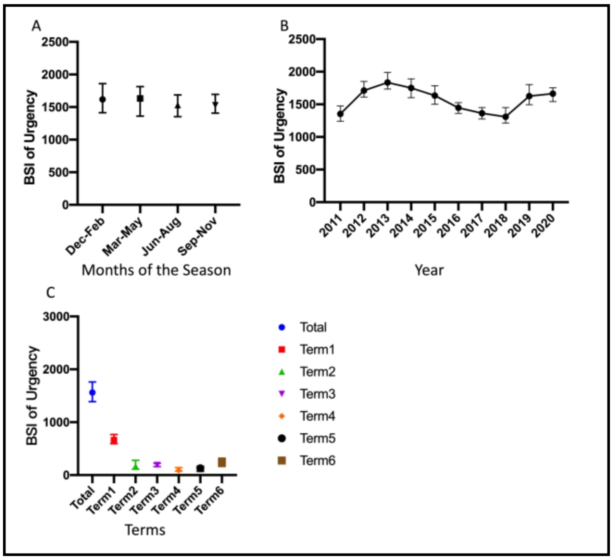

Urgency

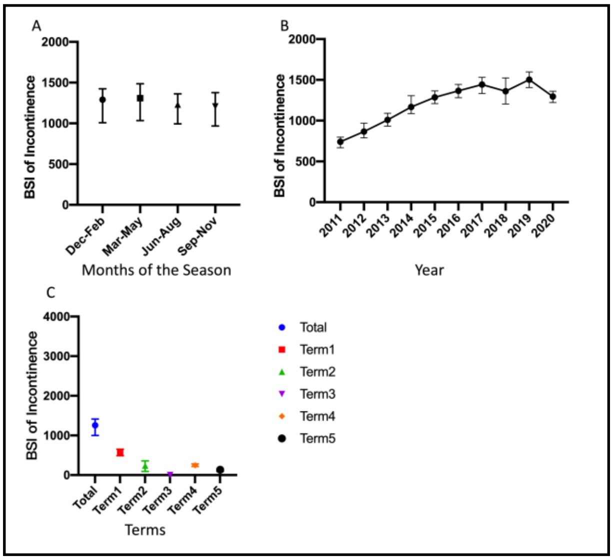

Incontinence

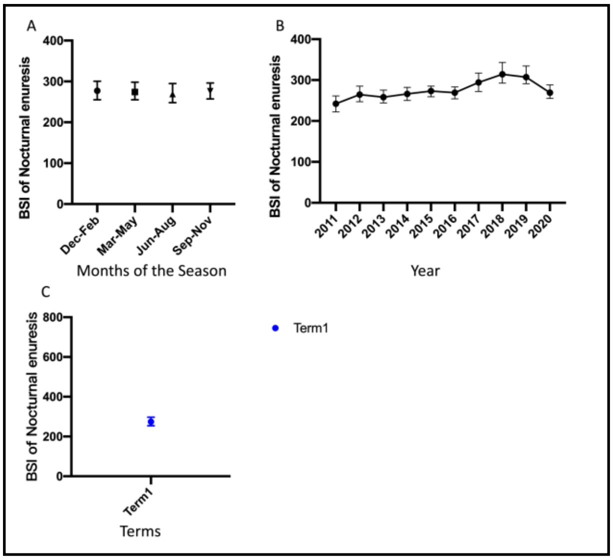

Nocturnal enuresis

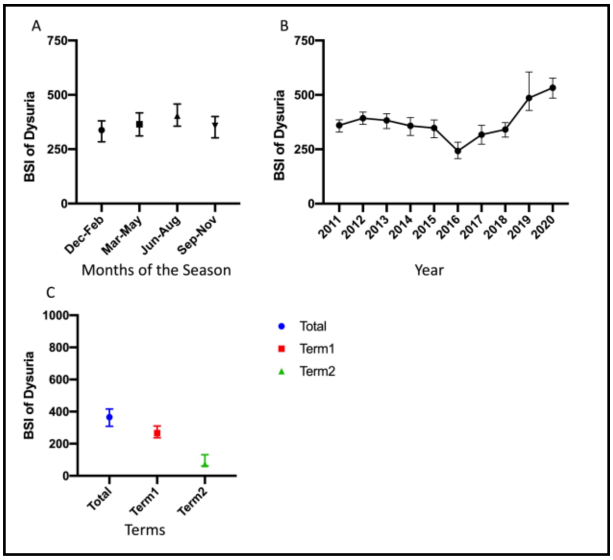

Dysuria

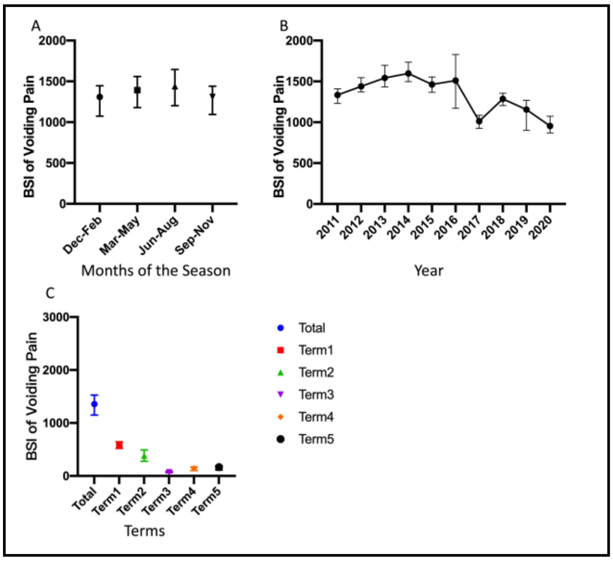

Voiding Pain

Supplimentary appendix 2. Web-based interest in LUTS domains over the last 10 years:
A: Median search volumes with IQRs for each term. **B:** Total BSI value (median [IQR]) for each season. **C:** Annual trend of the total BSI. **BSI:** Baidu search index; **LUTS:** lower urinary tract symptoms.
