# Supplementary material for: Using Search Trends to Analyze Web-Based Interest in Lower Urinary Tract Symptoms-Related Inquiries, Diagnoses, and Treatments in Mainland China: Infodemiology Study of Baidu Index Data
Source: J Med Internet Res. 2021 Jul 6;23(7):e27029. doi: 10.2196/27029 (PMC8292938; doi:10.2196/27029)
Supplement: Multimedia Appendix 5 [file jmir_v23i7e27029_app5.pdf]

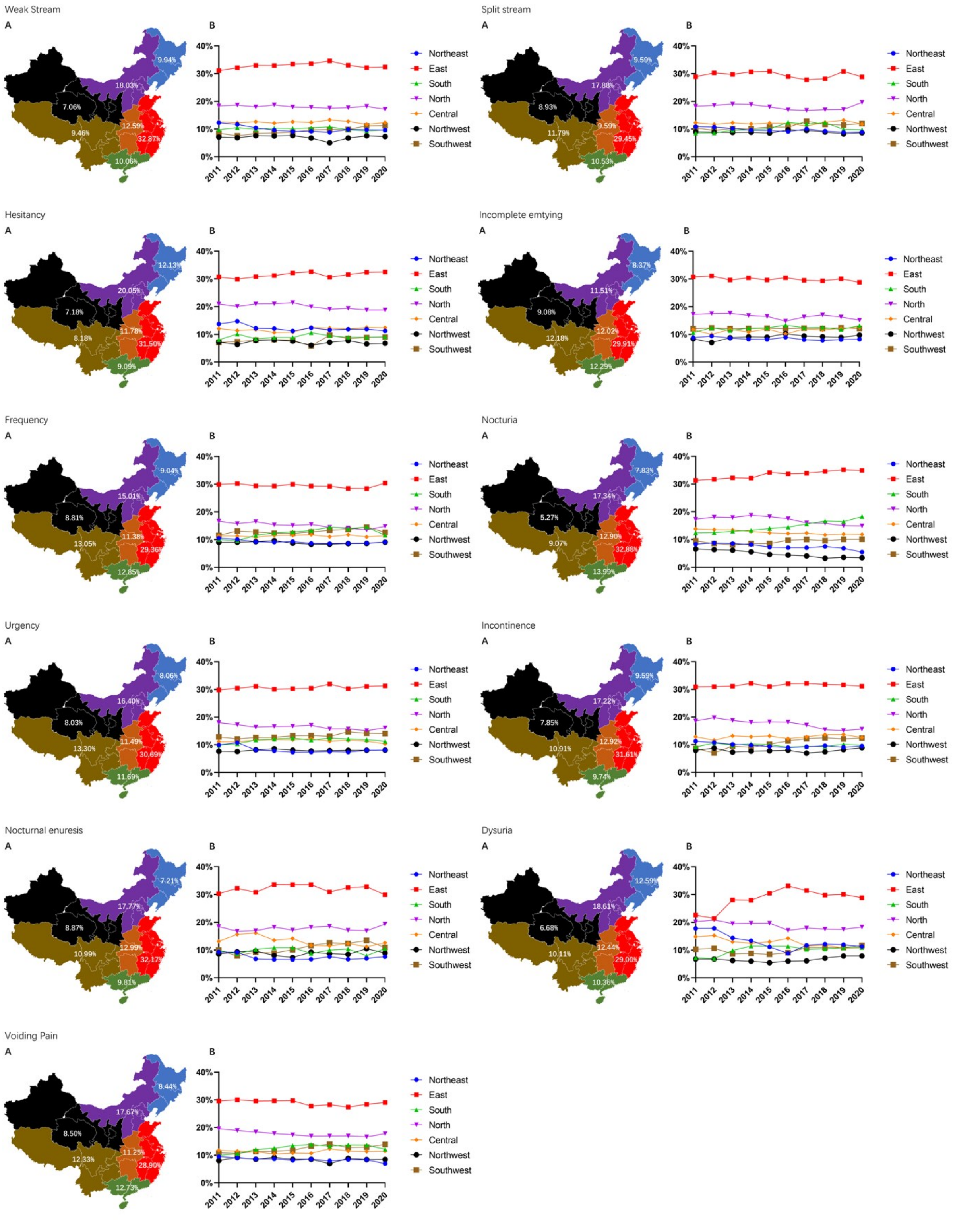

Regional distribution of online interest in each LUTS domain over the last 10 years.

A: Regional rates for each area. B: Annual trend of the BSI for each region.
