# Supplementary material for: Using Search Trends to Analyze Web-Based Interest in Lower Urinary Tract Symptoms-Related Inquiries, Diagnoses, and Treatments in Mainland China: Infodemiology Study of Baidu Index Data
Source: J Med Internet Res. 2021 Jul 6;23(7):e27029. doi: 10.2196/27029 (PMC8292938; doi:10.2196/27029)
Supplement: Multimedia Appendix 6 [file jmir_v23i7e27029_app6.pdf]

Domain/Category		Term1	BSI	Term2	BSI	Term3	BSI
Weak Stream	1	尿不尽	666,924	尿分叉	410,682	尿频尿急尿不尽	408,274
		incomplete emptying		urine split		frequency urgency and incomplete urine	
	2	尿不尽是怎么回事	860,814	尿分叉是怎么回事	185,294	尿频尿急尿不尽是什么原因造成的	153,316
		Why is incomplete emptying		Why is urine split		What causes frequency urgency incomplete	
	3	前列腺增生怎么治疗	60,576	前列腺炎吃什么药	57,144	前列腺炎吃什么药最好	46,172
		How to treat BPH		What medicine to take to cure prostatitis		What is the best medicine for prostatitis	
	4	利尿的食物	14,508	我的身体有 BUG	7,308	小便	7,240
		Diuretic food		I have physical BUGs		Urine	
	5	前列腺炎	1,500,686	膀胱炎	86,428	尿路感染	41,660
		Prostatitis		Cystitis		UTI	
	6	郑州颐和医院	3,522	泰州市人民医院	2,558	博爱医院是正规医院吗	2,478
		Yihe hospital of zhengzhou		City people hospital of Taizhou		Is the bo'ai hospital legal?	
	7	前列腺炎有什么症状	831,882	前列腺增生的症状	104,442	前列腺炎症状	12,976
		What are the symptoms of prostatitis?		What are the symptoms of BPH?		Symptoms of prostatitis	
	8	前列腺炎一杯水自测	24,688	前列腺检查	5,260	前列腺液检查	5,046
		Testing prostatitis with one cup of water		Prostatitis test		Prostatitis fluid test	
	9	前列腺炎能彻底治愈吗	21,728	前列腺炎会自愈吗	2,808	前列腺炎能根治吗	1,600
		Is prostatitis completely curable?		Is prostatitis self-healing		Is prostatitis curable?	
	10	肾气不足	8,026	肾气虚	5,854	湿热体质	2,584
		The insufficient Qi of the Shen (Kidney)		Qi deficiency of the Shen (Kidney)		damp heat constitution	
	11	湿热症状	844				
		Symptoms of damp heat constitution					
	12	知柏地黄丸	2,104				
		phellodendron and rehmannia bolus					
Split	1	尿不尽	1,131,818	小便刺痛	912,462	尿分叉	839,362
		Incomplete emptying		Stabbing pain urination		Urinary split	
	2	尿不尽是怎么回事	1,311,604	尿液特别黄是怎么回事	499,600	尿分叉是怎么回事	472,792
		Why is incomplete emptying		Why is urine yellow		Why is urinary split?	
	3	前列腺炎吃什么药	1,675,072	*"矮阳"*怎么恢复	1,573,308	怎么锻炼治早泄	489,278
		What's the medication		How to recover from "*Erecfile dysfuncsion*"		How to cure PE by training	
	4	什么是前列腺前列腺在哪	677,140	前列腺	489,094	*"包头"*过长手术*"包头"*过长	386,558
		What and where is the prostate		Prostate		Surgery for redundant "*prepuse*"	

	5	前列腺炎	6,086,456	包头过长	87,778	尿道狭窄	49,388
		Prostatitis		Redundant prepuce		urethral stricture	
	6	老虎油	11,744	男性医院	4,428	德胜门中医院	3,408
		Tiger balm		Andrology Hospital		TCM hospital of Desheng gate	
	7	前列腺炎有什么症状	26,683,978	前列腺增生的症状	367,976	尿道炎症状表现	66,268
		What are the symptoms of prostatitis?		What are the symptoms of BPH?		What are the symptoms of urethritis?	
	8	前列腺炎一杯水自测	2,288,702	前列腺按摩	458,324	男人常规检查	283,486
		Testing prostatitis with one cup of water		Prostate massage		Routine exam for males' (problem)	
	9	前列腺年轻人会自愈吗	1,126,664	长期过度手淫多久恢复	429,106	前列腺炎有什么症状和危害性	159,116
		Is prostatitis self-healing		How long it takes to recover after long-term excessive masturbating		What are the symptoms and damages of prostatitis	
	10	肾亏	60,744				
		Asthenia of Kidney-Yin					
	11	阳虚症状	3,898	脚心发热是怎么回事	1,664	肾阴虚症状	1,406
		Yang deficiency symptoms		why is the heat of the foot arch		Symptom of kidney-yin deficiency	
	12	穿山甲粉	1,832	枸杞有什么作用	1,416	陈皮的作用与功效	294
		Pangolin powder		What is the medication effect of goji berry		Medication effect of tangerine peel	
Hesitancy	1	尿频	819,376	尿不尽	620,294	尿急	433,758
		Urinary frequency		Incomplete emptying		Urinary urgency	
	2	尿不尽是怎么回事	131,044	尿分叉是怎么回事	71,902	尿滴白是什么原因	2,950
		Why is urinary frequency		Why is split stream		What cause the terminal white dribbling	
	3	前列康	51,856	前列腺增生怎么治疗	20,336	保列治	19,762
		Prostate Plus		How to cure BPH		Proscar	
	4	前列腺	155,796	括约肌	9,802	久坐的危害	7,876
		Prostate		Sphincter		risks of being seated for too long	
	5	前列腺炎	3,658,210	前列腺癌	99,886	膀胱炎	47,090
		Prostatitis		Prostate cancer		Cystitis	
	6	南京同仁医院	3,428	武汉中医院	1,550	益阳市中心医院	1,530
		Tongren hospital, Nanjing		TCM hospital,Wuhan		Central hospital,Yiyang	
	7	前列腺炎有什么症状	8,303,102	前列腺增生的症状	191,756	前列腺炎症状	79,432
		What are symptoms of the prostatitis		Symptoms of BPH		Symptoms of prostatitis	
	8	前列腺炎一杯水自测	484,170	前列腺液	14,900	前列腺按摩	14,528
		Testing prostatitis with one cup of water					
	9	前列腺年轻人会自愈吗	173,586	前列腺炎会自愈吗	76,674	前列腺炎能彻底治愈吗	30,292

		Is prostatitis self-healing in young men		Is prostatitis self-healing		Is prostatitis complete curable?	
	10	肾透支	978				
		renal physically overdraft exhausted					
	11	肾气不足的症状	10,268				
		Symptoms of kidney deficiency					
	12	葛根	310				
		The root of kudzu vine					
Incomplete Emptying	1	尿频	3,014,686	尿不尽	1,289,546	尿频尿急尿不尽	1,020,910
		Urinary frequency		Incomplete emptying		Frequency,urgency,incomplete emptying	
	2	尿频是怎么回事	6,751,142	尿频尿急是怎么回事	6,620,776	尿不尽是怎么回事	1,878,530
		Why is frequency		Why is frequency and urgency		Why is incomplete emptying	
	3	前列腺炎吃什么药	1,785,180	尿路感染吃什么药	1,475,304	三金片的功效与作用	1,102,312
		What medication to take for prostatitis		What medication to take for UTI		Efficacy of Sanjin tables	
	4	前列腺	393,966	人体正常体温是多少度	148,190	憋尿	59,120
		Prostatitis		What is the normal temperature of Human		Suppresses the urine	
	5	前列腺炎	6,083,440	尿路感染	932,908	膀胱炎	394,938
		Prostatitis		UTI		Cystitis	
	6	问医生	83,958	好大夫在线	74,466	拇指医生	6,130
		Ask Doctor.com		Good doctor.com		Dr thumb.com	
	7	前列腺炎有什么症状	22,680,122	尿毒症早期症状	3,816,456	尿道炎症状表现	1,545,902
		What are the symptoms of prostatitis		What are the early symptoms of uremia		Symptom of urinary tract inflammation	
	8	前列腺炎一杯水自测	699,184	自己按摩前列腺十种手法图解	114,014	妇科检查	15,334
		Testing prostatitis with one cup of water		Demonstration by illustration,10 ways of DIY prostate examination		Gynecologic examination	
	9	前列腺炎能彻底治愈吗	772,360	女性尿道感染能自愈吗	378,462	早些泄能治好吗	88,816
		Is prostatitis complete curable?		Is female UTI self-healing?		Is premature ejaculation curable?	
	10	肾虚	41,756	阴虚	19,200	阳虚	13,436
		deficiency of kidney energy		deficiency of YIN (negative)		deficiency of YANG (positive)	
	11	阴虚和阳虚最简单辨别	133,548	肾阴虚和肾阳虚的区别	70,654	肾阴虚的症状	39,842
		Simplest way in distinguish deficiency of YIN and YANG		Distinguish the deficiency of kidney YIN and YANG		Symptom of YIN deficiency of kidney	
	12	知柏地黄丸	39,206				

		phellodendron and rehmannia bolus					
Frequency	1	尿频	7,003,756	女性尿完一会儿又想尿	2,747,294	尿频尿急尿不尽	2,251,042
		Urinary Frequency		Women want to pee just after urinating		Freuqency,Urgency and incomplete emptying	
	2	尿频是怎么回事	17,391,286	尿频尿急是怎么回事	3,060,924	尿路感染是怎么引起的	2,627,612
		Why is urinary frequency		Why is frequency and urgency		How is UTI caused	
	3	三金片的功效与作用	7,772,934	尿路感染吃什么药	5,198,314	左氧氟沙星	4,053,408
		Efficacy of Sanjin pill		What medicine to take with UTI		Levofloxacin	
	4	前列腺	492,858	人体器官结构五脏六腑肾位置	186,472	肚子里咕噜咕噜响是怎么回事	164,050
		Prostatitis		Anatomic location of the five viscera and six entrails in human		What is rumble in the stomach	
	5	前列腺炎	6,500,962	尿路感染	4,353,686	糖尿病	1,997,930
		Prostatitis		UTI		Diabetes	
	6	好大夫	70,970	心理医生	35,494	广东省人民医院	33,788
		Good doctors.com		Psychiatrist.com		Guangdong provincial hospital	
	7	前列腺炎有什么症状	24,580,186	糖尿病的早期症状	9,532,584	尿毒症早期症状	5,689,434
		What are the symptoms of prostatitis		Early symptoms of diabetes mellitus		Early symptoms of uremia	
	8	前列腺炎一杯水自测	248,938	尿常规	88,818	验孕棒怎么用	69,336
		Testing prostatitis with one cup of water		Routine urinary test		How to use the pregnancy test stick	
	9	女性尿道感染能自愈吗	1,025,440	前列腺炎能彻底治愈吗	324,842	阳痿会自己恢复吗	90,596
		Is female UTI self-healing?		Is prostatitis complete curable?		Is impotence self-healing?	
	10	肾虚	264,352	肾阳虚	30,162	气血不足	27,978
		Deficiency of kidney energy		deficiency of kidney Yang		deficiency of Qi and blood	
	11	肾虚的表现	2,964,302	脾虚的症状	138,500	肾阴虚和肾阳虚的区别	104,914
		Manifestation of kidney energy deficiency		Manifestation of Spleen energy deficiency		Difference of kidney energy deficiency with essence of Yin and Yang	
	12	桂圆的功效与作用	99,762	葛根	79,870	夏枯草的功效与作用	78,344
		Efficacy of longan		The root of kudzu vine		Efficacy of prunella vulgaris	
Urgency	1	尿频	2,107,120	尿不尽	1,193,222	憋尿	1,087,090
		Urinary frequency		Incomplete		Suppresses the urine	
	2	尿频是怎么回事	4,112,752	尿路感染是怎么引起的	1,162,970	尿不尽是怎么回事	1,287,212
		Why is urinary frequency		How is the UTI caused		Why is the incomplete urinate	
	3	三金片	3,904,594	尿路感染吃什么药	2,502,692	六味地黄丸的功效与作用	2,210,286

		Sanjin pills		What's the medication for UTI		The efficacy of Liuwei Dihuang Pills	
	4	女性生殖	1,376,070	前列腺	387,986	幽门螺旋杆菌	250,894
		Female reproduce		Prostate		Helicobacter pylori	
	5	前列腺炎	2,145,816	尿毒症	669,734	尿路感染	528,024
		Prostatitis		Uremia		UTI	
	6	郑大一附院	21,930	丁香医生	20,476	春雨医生	113,24
		Fist affiliated hospital of Zhengzhou University		DXY.com		Dr Chunyu.com	
	7	前列腺炎有什么症状	13,278,380	尿毒症早期症状	2,824,338	怀孕 10 天最明显的征兆	1,636,842
		What are the symptoms of prostatitis		What are the early symptoms of Uremia		Most significant signs of the first 10days of pregnancy	
	8	前列腺炎一杯水自测	480,412	尿常规能检查出什么	39,822	体检项目有哪些	30,284
		Testing prostatitis with one cup of water		What can be detected by routine urinary test		What the items in physical examination	
	9	女性尿道感染能自愈吗	463,322	前列腺炎能彻底治愈吗	430,102	前列腺炎有什么症状和危害性	69,566
		Is female UTI self-healing		Is prostatitis completely curable?		What are the symptoms and damages for prostatitis	
	10	肾虚	116,188	肾阳虚	42,292		
		Deficiency of kidney energy		Deficiency of kidney Yang energy			
	11	肾阴虚表现	953,638	肾虚的表现	131,290	阴虚和阳虚最简单辨别	118,494
		Manifestation of deficiency of kidney Yin energy		Manifestation of deficiency of kidney energy		Distinguishing from Yang deficiency and Yin deficiency	
	12	黄芪红枣茶	1,172,242	蒲公英茶的功效与作用	661,598	车前草的功效与作用	248,128
		Jujube Huangqi tea		Dandelion Tea		The efficacy of plantain seed	
Dysuria	1	尿不尽	507,706	尿潴留	405,692	尿分叉	233,416
		Incomplete emptying		Urinary retention		Stream split	
	2	尿不尽是怎么回事	215,360	为什么尿不尽	57,052	尿不尽是什么引起	27,396
		Why is incomplete emptying		Why is incomplete emptying		What caused incomplete emptying	
	3	前列腺增生怎么治疗	336,008	尿结石最快的排出方法	98,584	前列腺炎的自我疗法	70,390
		How to treat BPH		The most effective way to lithagogue		DIY treatment of prostatitis	
	4	尿	119,762	小便	34,704	利尿的食物	29,930
		Urinate		Pee		Diuretic food	
	5	前列腺炎	163,268	尿路结石	106,928	尿结石	73,062
		Prostatitis		Lithangiuria		Urolithiasis	

	6	宝鸡市中心医院	2,410	温岭市第一人民医院	1,602	内蒙古国际蒙医医院	1,226
		Central hospital of Baoji City		First hospital of Wenling city		International hospital of Inner-Mongolia	
	7	前列腺增生的症状	731,558	尿结石的症状	88,710	膀胱炎有什么症状	51,954
		Symptoms of BPH		Symptoms of Urolithiasis		Symptoms of cystitis	
	8	前列腺炎一杯水自测	98,684	膀胱镜	7,056	直肠指检	1,626
		Testing prostatitis with one cup of water		Cystoscope		Digital rectal examination	
	9	前列腺年轻人会自愈吗	17,406	尿结石严重吗	620	肾结石的危害	500
		Is prostatitis self-healing?		Is Urolithiasis leading sever consequence		Damage of urolithiasis	
	10	膀胱经不通	220				
		Obstruction of urinary Bladder lines					
	11	肾亏的症状	228				
		Manifestation of kidney energy deficiency					
	12	葛根	70				
		The root of kudzu vine					
Nocturnal enuresis	1	遗精	1,314,062	尿失禁	469,544	尿不尽	446,866
		spermatorrhea		Urinary incontinence		Incomplete emptying	
	2	尿不尽是怎么回事	165,026	屁多是什么原因	55,582	遗精是什么原因引起的	45,644
		Why is incomplete emptying		What caused farting too much		What caused spermatorrhea	
	3	醒脾养儿颗粒	92,542	阿米替林	32,538	小儿推拿	22,000
		Feed spleen nourish kid particle		Amitriptyline		Massage for kid	
	4	泌尿外科	27,468	感统失调	20,416	母乳喂养多久最好	19,088
		Dep of Urology		Sensory dys-integration		how long of breastfeeding is the best	
	5	多动症	236,148	尿崩症	192,550	脊柱裂	141,434
		attention deficit hyperactivity disorder (ADHD)		diabetes insipidus		spina bifida	
	6	南京儿童医院	50,484	21 金维他	21,264	儿童医院	16,146
		Children's hospital Nanjing city		21 gold vitamines		Children's hospital	
	7	多动症有哪些症状	95,164	尿道炎症状表现	48,944	多动症儿童的行为表现有哪些	27,588
		Symptoms of ADHD		Symptoms of urinary tract inflammation		Symptoms of ADHD in children	
	8	试纸	4,438	膀胱镜	2,996	PPD 试验	2,096
		Test paper		cystoscope		PDD test	
	9	糖尿病会遗传吗	4,334	糖尿病遗传吗	2,806	蛛网膜下腔出血后遗症	1,036
		Can diabetes mellitus hereditary		Is diabetes mellitus hereditary		Sequelae of subarachnoid hemorrhage	
	10	肾气不足	8,626	疳积	7,406	肾气虚	5,842

		Kidney Qi deficiency		infantile malnutrition		Kidney Qi weak	
	11	肾气不足的症状	13,406	肾亏的症状有哪些	12,398	脾肾阳虚的症状	6,996
		Symptoms of kidney Qi deficiency		Symptoms of kidney weakness		Symptoms of kidney Spleen Yang deficiency	
	12	山药	54,210	分心木	14,940	金樱子	12,290
		Chinese yam		Diaphragma juglandis		Rosa laevigata	
Nocturia	1	尿频	2,103,352	尿不尽	398,566	尿多	264,042
		Urinary frequency		Incomplete emptying		Polyuria	
	2	尿频是怎么回事	606,1632	夜尿多是怎么回事	1,388,138	尿频尿急是怎么回事	300,240
		Why is urinary frequency		Why is nocturia		Why is frequency and urgency	
	3	六味地黄丸的功效与作用	6,298,692	金匮肾气丸的功效与作用	3,779,026	三金片的功效与作用	1,208,160
		Efficacy of Liuweidihuang pill		Efficacy of kidney-qi-tonifying pill		Efficacy of Sanjin pill	
	4	芒果的功效与作用禁忌	697,896	指甲上有竖纹是怎么回事	331,576	前列腺钙化是什么意思	170,444
		Efficacy and taboo of mango		What is the sign of vertical grain on nail		What is prostatic calcification	
	5	前列腺炎	1,069,872	尿毒症	851,004	肾炎	388,944
		Prostatitis		Uremia		Nephritis	
	6	好大夫在线	78,668	健康之路	43,214	郑大一附院	25,124
		Good doctor.com		Health road.com		First affiliated hospital of Zhengzhou University	
	7	前列腺炎有什么症状	12,504,694	尿毒症早期症状	5,699,300	糖尿病的早期症状	3,621,064
		What are the symptoms of prostatitis		Early signs of uremia		Early signs of diabetes mellitus	
	8	前列腺炎一杯水自测	242,412	肾功能检查项目	109,974	24 小时尿蛋白定量	81,616
		Testing prostatitis with one cup of water		Testing items of renal function		24 hours urinary protein quantification	
	9	女性尿道感染能自愈吗	30,894	尿毒症能活多久	26,328	前列腺炎会自愈吗	25,442
		Is female UTI self-curing		How long to survive with uremia		Is prostatitis self-curing	
	10	肾阳虚	273,698	肾虚	177,190	肾阴虚	73,646
		Kidney deficiency		Kidney deficiency		Kidney Yin-deficiency	
	11	肾虚的表现	1,457,538	阴虚和阳虚最简单辨别	366,450	肾阴虚和肾阳虚的区别	355,376
		Manifestation Kidney deficiency		Distinguish from Yang-Yin-deficiency		kidney Yang-Yin-deficiency differences	
	12	五苓散	20,736	黄芪的功效与作用	525,134	桑葚的功效与作用	441,114
		Wuling powder		Efficacy of Astragalus		Efficacy of mulberries	
Incontinence	1	尿急	895,220	尿潴留	630,182	尿失禁	537,402
		Urgency		Urinary retention		Urinary incontinence	
	2	女性漏尿是什么原因	202,706	尿路感染是怎么引起的	172,708	尿频是怎么回事	107,608

		Why is female urinary leakage		What caused UTI		Why is urinary frequency	
	3	凯格尔运动	1,928,366	盆底肌修复	139,300	米多君	46,368
		Kegel Exercise		Pelvic floor muscle repair		Midodrine	
	4	前列腺	152,898	膀胱	80,806	幽门螺杆菌	68,832
		Prostate		Bladder		Helicobacter pylori	
	5	尿路感染	438,592	子宫脱垂	209,622	压力性尿失禁	206,794
		UTI		Uterus prolapse		Stress incontinence	
	6	好大夫	106,878	平安好医生	62,512	养生堂	27,290
		Good doctor.com		Ping'an Doctor.com		Honyaradoh	
	7	膀胱炎有什么症状	322,618	尿毒症早期症状	320,342	尿路感染症状	259,826
		What are the manifestation of cystitis		Early symptoms of uremia		Symptosm of UTI	
	8	血小板	24,046	肌酐正常值	15,186	妇科检查	12,242
		platelet		Normal range or creatine		Gynecology exame	
	9	女性尿道感染能自愈吗	77,960	肾积水严重吗	8,206	脑血栓后遗症	4,308
		Is female UTI self-healing		Is hydronephrosis server		cerebral thrombosis sequel	
	10	NG					
		NG					
	11	夜尿多是怎么回事	8,796	肾气不足的症状	3,484		
		Why is nocturia		Symptom of insufficient Qi in kidney			
	12	肾蕨	2,312	鳖精	723		
		tuberous sword fern rhizome		essence of turtle			
Voiding Pain	1	尿频	1,676,980	尿道内刺痛	1,599,580	尿频尿急尿不尽	568,856
		Urinary frequency		Pain in urinary tract		Freuqency,urgency,incomplete emptying	
	2	尿频是怎么回事	4,910,316	尿路感染是怎么引起的	2,682,264	小便时尿道刺痛是怎么回事	1,633,620
		Why is urinary frequency		Why is UTI		Why urinary tract Pain	
	3	三金片的功效与作用	5,455,266	左氧氟沙星	5,048,482	尿路感染吃什么药	4,469,254
		Efficacy of Sanjin pill		Levofloxacin		What medication for UTI	
	4	前列腺	767,236	耻骨是哪个部位	167,126	膀胱	143,326
		Prostate		Position of pubic bone		Bladder	
	5	前列腺炎	2,132,004	尿路感染	1,130,530	尿道炎	795,664
		Prostatitis		UTI		Urethritis	
	6	上海市第一人民医院	10,206	云南省第一人民医院	7,694	快速问医生	6,922
		First hospital of Shanghai city		First hospital of Yunnan province		Doctor quick.com	
	7	前列腺炎有什么症状	16,312,850	尿毒症早期症状	6,609,454	尿道炎症状表现	1,949,984

	Symptoms of prostatitis		Early symptoms of uremia		Symptoms of urinary tract inflammation	
8	前列腺炎一杯水自测	62,044	HCG 值多少是怀孕	56,266	血常规能检查出什么	48,576
	Testing prostatitis with one cup of water		HCG value indicating pregnancy		What can be detected by Blood routine test	
9	女性尿道感染能自愈吗	1,385,942	前列腺炎能彻底治愈吗	178,722	阳痿会自己恢复吗	52,228
	Is female UTI self-curing		Is prostatitis completely curable?		Is impotence self-recovering	
10	肾阳虚	47,044				
	kidney-Yang deficiency					
11	肾虚的表现	251,026	肾阳虚和肾阴虚的区别	120,592		
	Manifestation of kidny deficiency		Difference between kidney Yang deficiency and Yin deficiency			
12	黄芪的功效与作用	379,838	蒲公英	184,088	白术的功效与作用	107,080
	Efficacy of astragalus		dandelion		Efficacy of atractylodes	
